# Optimizing PrEP Continuance: A Secondary Analysis Examining Perceived Autonomy Support and Care Coordination Quality among Black MSM in HPTN 073

**DOI:** 10.3390/ijerph19084489

**Published:** 2022-04-08

**Authors:** S. Raquel Ramos, Geetha Beauchamp, Darrell P. Wheeler, Leo Wilton, Darren L. Whitfield, Donte T. Boyd, Lisa Hightow-Weidman, Sheldon D. Fields, LaRon E. Nelson, on behalf of the HPTN 073 Team

**Affiliations:** 1 School of Nursing, Yale University, Orange, CT 06477, USA; laron.nelson@yale.edu; 2 Department of Social and Behavioral Sciences, School of Public Health, Yale University, New Haven, CT 06520, USA; 3 Center for Interdisciplinary Research on AIDS, School of Public Health, Yale University, New Haven, CT 06520, USA; 4 SCHARP, Fred Hutchinson Cancer Research Center, Seattle, WA 98109, USA; geetha@scharp.org; 5 Office of the Provost, Iona College, New Rochelle, NY 10801, USA; dwheeler@iona.edu; 6 Department of Human Development, State University of New York at Binghamton, Binghamton, NY 13901, USA; lwilton@binghamton.edu; 7 School of Social Work, University of Maryland, Baltimore, MD 21201, USA; darren.whitfield@ssw.umaryland.edu; 8 College of Social Work, The Ohio State University, Columbus, OH 43210, USA; boyd.465@osu.edu; 9 Department of Medicine, University of North Carolina, Chapel Hill, NC 27599, USA; lisa_hightow@med.unc.edu; 10 College of Nursing, Pennsylvania State University, University Park, PA 16803, USA; sheldondei@psu.edu; 11 MAP Centre for Urban Health Solutions, Unity Health Toronto, St. Michael’s Hospital, Toronto, ON M5B 1W8, Canada

**Keywords:** HIV, PrEP, Black MSM, patient care management, decision making, autonomy support, care coordination, healthcare quality

## Abstract

At the end of year 2018, it was estimated that in the United States over 1 million people were living with HIV. Although Black/African American individuals comprise an estimated 13.4% of the US population, as of 2019, they represented an estimated 42% of all new HIV diagnoses in 2018. PrEP use among Black men who have sex with men has not reached levels sufficient to have a population impact on HIV incidence. The purpose of this study was to examine whether high perceived autonomy support and care coordination quality were associated with PrEP continuation. Secondary analyses were conducted on data with 226 Black MSM in three US cities. Participants who were PrEP users and scored higher on autonomy support at week 8 were significantly more likely to continue PrEP (OR 1.48; 95% CI 1.04–2.11). Perception of coordination quality did not differ between PrEP users and non-users at any of the visits. Although coordination quality was not statistically significant, greater than half of PrEP users and non-PrEP users utilized the C4 services. Addressing social, individual, and structural barriers to PrEP may benefit Black MSM irrespective of their PrEP use.

## 1. Introduction

In the United States (US), it was estimated that 1.2 million people were living with human immunodeficiency virus (HIV) at the end of year 2018 [1]. Although Blacks/African Americans comprise an estimated 13.4% of the US population, as of 2019 [2], they represented an estimated 42% of all new HIV diagnoses in 2018 [1]. 

In 2012, pre-exposure prophylaxis (PrEP), a highly effective medication, was approved by the US Food and Drug Administration (FDA) for HIV prevention [3]. It’s original formulation, emtricitabine/tenofovir (FTC/TDF), administered as a once-a-day pill, has been approved for all adolescents and adults at risk of HIV through sexual exposure or injection drug use [3]. In 2019, the FDA approved an additional PrEP medication for HIV prevention, emtricitabine and tenofovir alafenamide (TAF-FTC), known as Descovy, in all adolescents and adults except persons who engage in receptive vaginal sex [4]. Taking PrEP as prescribed, is safe, cost-effective, and can reduce HIV acquisition by over 99% [5]. However, all persons eligible for PrEP are not aware of its availability. 

Findings from an analysis of national data analyzed between 2014–2017, suggested that awareness of pre-exposure prophylaxis (PrEP) increased, and that uptake of PrEP increased as well [6]. The increase in PrEP use was most significant in white men who have sex with men (MSM) when compared with Black MSM and was associated with income, education, and health care visits [6]. However, 2018 epidemiological data suggests that the highest HIV incidence is in Black MSM populations in the South, Midwest, and Northeast [1]. The majority of Americans estimated to benefit from PrEP are Black; yet Black individuals only represent a small proportion of those taking PrEP [7,8,9]. PrEP use among Black MSM has not reached levels sufficient to have a population impact on HIV incidence. This is in large part due to the documented structural inequities in health care that impact PrEP accessibility and engagement [10,11,12].

HIV Prevention Trials Network (HPTN) protocol 073 [13], was a vanguard pioneering study conducted from 2013 to 2015 that offered PrEP and a culturally sensitive novel autonomy-supportive approach to service provision, called client-centered care coordination (C4^TM^) (herein referred to as care coordination) [14,15] to increase PrEP uptake and adherence among Black MSM. C4^TM^ was a structured, theory-based, autonomy-supportive approach to behavioral counseling and activities to address unmet psychosocial and health systems barriers to HIV risk reduction [14,16]. Components of care coordination included a comprehensive assessment of factors (clinical, social, and structural) influencing health, providing intensive behavioral counseling based on self-determination theory, and coordination of intersectional services to meet client-identified needs, including referrals for sexual health, mental health, and legal support services [17]. Care coordination was used to engage clinicians and study staff in supporting Black MSM’s progression along the PrEP cascade [18]. HPTN 073 enrolled (*n* = 226) Black MSM who were eligible for PrEP [13]. PrEP usage was not a condition for study enrollment, nor was it required for participation. Although many participants initiated PrEP at some point during the study, we were interested in the relationship between perceptions of autonomy supportive care and PrEP continuation. We will examine secondary data on care coordination, focus of care coordination encounters, frequency and duration of the encounters, and the complexity level of the encounters to provide context on how this may impact perception of autonomy support and care coordination quality. The purpose of this study was to examine whether perceived autonomy support and care coordination quality were associated with PrEP continuation among Black MSM who initiated PrEP in HPTN 073. We hypothesized that high perceived autonomy support and high perception of coordination quality are negatively associated with PrEP continuation.

## 2. Materials and Methods

### 2.1. Parent Study Design, Setting, and Recruitment

In HPTN 073, (*n* = 226) Black MSM who were HIV negative were recruited from Chapel Hill, North Carolina, Washington, DC, and Los Angeles, California to participate in a multidimensional care coordination intervention over the course of 52-weeks. A secondary analysis of data was conducted of Black MSM PrEP users (*n* = 178) and non-PrEP users (*n* = 48) in the HPTN 073 sample.

Briefly, data were collected from 2014 to 2017. Participants in the parent study were recruited in a manner that protected the rights and privacy of interested persons. Recruitment consisted of clinic referrals from partnering organizations, venue-based recruitment (local bars, community-based organizations, events, etc.), referrals from existing participants, community members, and health care providers. Additionally, based on study location, local radio and newspaper advertisements, and social media sites were utilized, all of which were targeted to Black MSM. Participants had varied site specific incentives based on attendance to their regularly scheduled visits and completion of measures, completion of all study visits, and a post study follow-up. Inclusion criteria was extensive. A few of the criteria included: (1) ages 18+; (2) no prior HIV diagnosis; (3) male sex at birth; (4) at high risk for HIV (as indicated by sub-criteria) and other criteria that can be found elsewhere [16,17,19].

The parent study and all procedures were approved by the institutional review boards of the University of California, Los Angeles (IRB#13-000422), University of North Carolina at Chapel Hill and Wake County Health and Human Services (IRB#13-1556), and George Washington University (IRB#041327). The study was also registered with DAIDS Document ID#11894 and ClinicalTrials.gov Identifier NCT01808352. Full details on the study protocol, parent HPTN 073 study, and C4^TM^ intervention can be found on clinicaltrials.gov and published elsewhere [16,17,19]. In this paper, present findings from a secondary analysis using a deidentified dataset from the parent HPTN 073 study.

### 2.2. Data Collection

**Care Coordination Measurement Log.** Research team members trained as care coordinators completed a care coordination measurement log (CCML) using a case report form (CRF) adapted from a publicly available care coordination measurement tool used in previous studies [20,21]. The CCML is an audit log that catalogs what activities or interactions were completed with the client during the visit. It included standardized instructions for how to document the characteristics of the care coordination encounter. Completion of the CCML could occur at standard study visits or in the interim between study visits. A CCML was not completed if no care coordination activity occurred or if the amount of time expended by the care coordinator to complete the activity was less than five minutes.

The individuals trained to provide care coordination had a range of occupations that included: (1) counselors, (2) nurse practitioners, (3) social workers, (4) registered nurses, (5) physician assistants, (6) marriage and family therapists, and (7) HIV test counselors. Use of the CCML CRF was triggered anytime the research team member performed care coordination activities that required more than five minutes to complete. Self-reported survey data were also collected from enrolled Black MSM using audio-computer assisted self-interview (ACASI).

**Focus of the Care Coordination Encounter.** Care coordination encounters focused on 10 areas: (1) mental health, (2) employment, (3) substance use, (4) legal issues, (5) sexual health, (6) referral management, (7) clinical management, (8) social services, (9) PrEP support, and (10) linkage to HIV care. Although there could have been more than one issue presented, a single focus was documented for the encounter by the coordination counselor.

**Frequency and Duration of Care Coordination Encounters**. Care coordination frequency was operationalized as the total number of minutes (starting from 5 min or more) the counselor spent on activities to address a participant’s health or social need that required them to engage in direct contact with a third person. Care coordination duration was assessed using the total number of care coordination encounters that require five or more minutes of care coordination. Screening visit sessions were treated as enrollment visits.

**Level of Complexity**. Care coordinators categorized the complexity of the client’s care coordination needs as one of four psychosocial levels, specifically assessing immediate risk to PrEP adherence: (1) no immediate complicating issues; (2) minimal immediate complicating issues; (3) moderate immediate complicating issues and (4) serious immediate complicating issues.

### 2.3. Measures

Data on PrEP continuation were collected from enrolled Black MSM using self-reported measures at 13-, 26-, 39-, and 52-week follow-up visits. The data on HCCQ and CPCQ were collected using audio-computer assisted self-interview (ACASI). The duration (in minutes) to complete ACASI across all visits was 29 (IQR 20 to 43).

#### 2.3.1. Predictor Variables

**Perceived Autonomy Support**. Perceived autonomy support was measured using the Healthcare Climate Questionnaire (HCCQ) [22]. The HCCQ is a 15-item scale on a 7-point Likert measure with levels of agreement ranging from “strongly agree to strongly disagree”. Autonomy support was defined as the degree to which a patient perceives their providers were supportive about a health care issue. The participants were instructed to think of the term ‘provider’ as ‘healthcare team’. Higher scores correspond with a higher perception of autonomy support. The HCCQ was administered starting at week 4, and then at weeks 8, 13, 26, 39, and 52 allowing participants to have multiple experiences with their providers over the course of 52 weeks. Assessment of week 4 responses indicated very good internal consistency with Cronbach’s alpha of 0.90. Across all visits, the sum of the HCCQ scores ranged from 15–105 and the average of the 15-item scale ranged from 1.3 to 7. Higher mean scores correspond with a higher perception of autonomy support.

**Perception of Coordination Quality and Care Coordination**. Perception of coordination quality was measured using the Client Perception of Coordination Questionnaire (CPCQ) [23]. The CPCQ was a 15-item scale on a 5-point Likert scale measure and the levels of agreement ranged from “never to always”. The CPCQ was administered giving sufficient time to assess C4 encounters up to week 13, and then at weeks 26, 39, and 52. Assessment of week 13 responses indicated very good internal consistency with Cronbach’s alpha of 0.92. Across all visits, the sum of the CPCQ scores ranged from 15–75 and the average ranged from 1.0 to 5.0. Higher mean scores correspond with a higher perception of coordination quality. Care coordination sessions and time were defined as total number of minutes spent from a schedule study visit to the next visit, which included the interim visits.

#### 2.3.2. Outcome Variable

**PrEP Continuation**. Our outcome variable was PrEP continuation. PrEP continuation status was assessed based on product (i.e., PrEP) hold CRF at each follow-up visit. PrEP users who were not on product hold or not permanently discontinued prior to that visit were considered continuing with PrEP use. Product hold could be initiated by clinician or by the study participant. Possible reasons for a product hold were: (1) an exit visit; (2) abnormal lab value; (3) clinical reasons determined by investigator; (4) Hepatitis B infection; (5) one of more reactive HIV results; (6) concern about HIV infection; (7) reported use of prohibited concomitant medication; (8) reported of use of post-exposure prophylaxis; (9) client requested temporary drug holiday; (10) client decided to terminate study regimen; (11) client is unwilling or unable to comply with required study procedures. The most applicable reason for permanent discontinuation or product hold was marked on the CRF.

## 3. Analysis

Characteristics of Care coordination frequency, duration, quality, and autonomy support were examined using Wilcoxon rank sum to compare the continuous variables and Chi-square was used to compare the categorical variables. The association between the binary outcome measure PrEP continuation (1 = PrEP continuation, 0 = on product hold or permanently discontinued) and perceived autonomy support at weeks 4 and 8, and week 13 CPCQ were separately assessed while adjusting for site. A longitudinal analysis was conducted using generalized estimating equation (GEE) with logistic link and exchangeable correlation matrix to account for the repeated measures for each participant. All analyses were conducted using SAS version 9.4 (SAS Institute, Cary, NC, USA).

## 4. Results

The sample consisted of 226 Black (African American, African, Afro-Caribbean, and Afro-Latino) MSM from three US cities. Seventy-nine percent of participants (*n* = 178) initiated PrEP throughout the course of the study.

### 4.1. Frequency and Duration of Care Coordination Encounters

At enrollment, the average duration of care coordination encounters (in minutes) for PrEP users was 48 (SD = 27) for PrEP users and 43 (SD = 23) for non-PrEP users (*p* = 0.61) (Table 1). The care coordination sessions were shorter during the follow-up visits, ranging from 7 to 31 min for non-PrEP users compared with PrEP users (18 to 24 min). Those who were on PrEP utilized a significantly higher number of minutes in care coordination at weeks 39 and 52. There was a statistically significant difference between PrEP users and non-users in the number of care coordination minutes at weeks 39 (*p* < 0.001) and 52 (*p* = 0.003) and in number of sessions at weeks 39 (*p* < 0.001) and 52 (*p* = 0.003) (Table 1).

### 4.2. Focus of Care Coordination Encounter

A total of 915 care cordination encounters services were provided from enrollment up to the 52-week follow-up period. The focus of the care coordination encounters ranged in terms of both topics being addressed and the frequency. For example, at enrollment, 59% of the visits were for social services (e.g., housing/food/insurance/transport, etc.)—data not shown. Among PrEP users, the highest percentages of encounters were for PrEP support (52%) and sexual health (17%), whereas among non-PrEP users the encounters were social services (33%), followed by sexual health (29%) (Table 2). The smallest proportion of encounters focused on legal/justice issues (<1%), and linkage to HIV care (<1%).

### 4.3. Level of Complexity

Of the *n* = 915 C4 encounters, the majority (*n* = 821) were documented as participants having no immediate psychosocial issues; 64 had minimal issues; 28 had moderate issues and 2 had serious issues. There was no statistical difference between PrEP users versus non-PrEP users when comparing those with no immediate psychosocial needs with those who had at least minimal immediate psychosocial needs (chi-square test, *p*= 0.20).

### 4.4. Autonomy Support on PrEP Continuation

Across all visits, the average autonomy support score was 6.17 (SD = 1.15). There was no difference between PrEP users and non-users at any of the visits (Table 3). There were no statistically significant differences in the mean score at week 4 compared with later visits (*p* = 0.26), and there were no differences in autonomy support between PrEP users and non-users from one visit to the other (*p* = 0.75).

Participants who were PrEP users and scored higher on autonomy support at week 8 were significantly more likely to continue with PrEP (OR 1.48; 95% CI (1.04, 2.11) (Table 4).

### 4.5. Perception of Coordination Quality on PrEP Continuation

Across all visits, the average perception of the care coordination quality score was 4.28 (SD 0.71) with a range of one to five. Perception of coordination quality did not differ between PrEP users and non-users at any of the visits (Table 3). There was no association between perception of coordination quality and PrEP continuation (Table 4).

## 5. Discussion

The purpose of this study was to examine whether high perceived autonomy support and care coordination quality were associated with PrEP continuation. This work is significant in advancing HIV prevention science as studies using national data found that although PrEP awareness had increased, PrEP uptake remained low in Black MSM [6]. Findings from this study suggested that perceived autonomy support and care coordination quality were positively associated with continuation of PrEP. Further, findings indicated that care coordination services were used equally by both those who initiated PrEP and those who did not use PrEP. This suggests that multicomponent interventions, such as HPTN 073, that use autonomy support and care coordination approaches can benefit Black MSM irrespective of their PrEP use.

The intervention used in HPTN 073 was a holistic approach to HIV prevention. It not only addressed HIV prevention by offering PrEP, but it also utilized other elements of care coordination, such as psychosocial support in hopes to amplify Black MSM in achieving their HIV prevention goals. As a component of care coordination encounters, our analysis of client complexity level data suggested that, overall, there presenting psychosocial issues among PrEP users and non-users did not require immediate intervention. The majority of PrEP users’ care coordination was for PrEP support, whereas that of non-PrEP users’ was for other services, including social services, sexual health services and medical referrals. PrEP non-users may have prioritized other urgent needs that were indirectly related to PrEP uptake, such as unstable housing, food insecurity, or other psychosocial needs.

Nonetheless, the provision of autonomy support was a valuable component to care coordination to address the motivational needs that influence Black MSM [24,25,26] and their uptake and/or continuation of PrEP. Future HIV prevention interventions should incorporate multiple elements of care coordination to enhance care, with special consideration given to the psychosocial factors influencing HIV inequalities among Black MSM [25,27]. This could be very influential for PrEP continuation when multiple care coordination services are offered and accessible.

Care coordination and autonomy support were provided to Black MSM over the course of one year. As a result, our findings suggested a notable decrease in the amount of time (in minutes) that was expended for participants in HPTN 073. The decrease in time may be related to an enhanced sense of self-efficacy that participants had in seeking out services and not needing as much support from the care coordinators. This sense of self-efficacy may have also facilitated PrEP continuation. Findings from a similar study corroborate that self-efficacy was a key component to health seeking behavior [28,29,30]. Additionally, our findings suggested that PrEP users who had greater autonomy support at week eight were more likely to continue PrEP. In an HIV medication adherence study [31], findings suggested that support from family and health care staff facilitated autonomous PrEP decision-making. The perception of having autonomy support from health care providers may be instrumental in the uptake and continuation of PrEP in Black MSM.

Perceived autonomy support and care coordination quality are important components of the C4^TM^ intervention. Based on the parent study intervention and our findings that with greater C4^TM^ use, visit frequency and visit intensity decreased and perceived autonomy support was advantageous to PrEP continuation, we surmise that directions for further research should focus on provider/patient communications and the bolstering of autonomy supportive care. Additionally, in order to increase awareness, uptake, and continuance of PrEP in Black MSM, interventions should focus on multiple component care coordination strategies that target Black MSM. This study has strong promise of addressing policy and increase HIV health equity if utilized as framework to scale up PrEP implementation in health care organization [32].

### Limitations

There were some limitations to this study. First, the data analyzed were from existing data and our analysis was limited to the variables in the dataset. However, Black MSM are underrepresented in HIV prevention research and our examination has advanced our understanding of how care coordination services can address their HIV prevention goals, specifically around PrEP continuation. Second, as with all survey data, responses are subject to recall and social desirability bias. It is possible that some participants may have chosen to limit or enhance their responses. However, the use of ACASI is best practice for the administration of survey measures that collect sensitive data [33] decreasing the possibility inaccurate participant responses. Third, we examined PrEP continuation within the context of autonomy support and care coordination quality, which are only a few of many influential factors, outside the clinical setting, that could affect an individual’s decision to continue or discontinue PrEP. However, we offered extensive, high quality care coordination to all participants decreasing the likelihood of continuation. Last, sample size may limit generalizability of the findings to the broader population of Black MSM. However, because we recruited from three US cities our findings provide cautiously generalizable insights into how autonomy support and quality care coordination can be positively associated with PrEP use in Black MSM from similar city demographics.

## 6. Conclusions

The purpose of this study was to examine whether high perceived autonomy support and care coordination quality were associated with PrEP continuation in Black MSM in HPTN 073. We found that the perception of having high autonomy support from health care providers was contributory PrEP use and continuation in Black MSM. We also found that the number and time in minutes of encounters decreased as participants engaged in care coordination and perceived high autonomy support from their provider. This may have resulted in higher-self efficacy and the persistence to continue PrEP. Quality care coordination and high, perceived autonomy support are conducive to PrEP continuation in Black MSM.

## Figures and Tables

**Table 1:** Care Coordination services by PrEP status and visit.

	Number of Visits Mean (SD)			C4 Minutes Mean (SD)		
	PrEP Users	PrEP Non-Users	*p*-Value ^1^	PrEP Users	PrEP Non-Users	*p*-Value ^1^
Enrollment	1.53 (0.82)	1.45 (0.72)	0.765	48 (27)	43 (23)	0.610
Week 4	0.63 (0.49)	0.60 (0.50)	0.650	18 (17)	15 (16)	0.352
Week 8	0.66 (0.52)	0.53 (0.51)	0.199	19 (18)	13 (14)	0.082
Week 13	0.73 (0.63)	0.70 (0.77)	0.581	22 (25)	21 (37)	0.187
Week 26	0.82 (0.62)	0.72 (0.61)	0.413	24 (21)	31 (48)	0.643
Week 39	0.74 (0.63)	0.27 (0.45)	<0.001	22 (21)	7 (12)	<0.001
Week 52	0.64 (0.57)	0.34 (0.48)	0.003	18 (19)	9 (13)	0.003
Overall	0.79 (0.66)	0.64 (0.67)	<0.001	23 (23)	19 (29)	0.006

^1^ Wilcoxon two-sample ranksome test.

**Table 2:** Care coordination focus by PrEP use from enrollment to 52 weeks follow-up.

	PrEP Users *n* = 178	Non-PrEP Users *n* = 48	Total
PrEP support	52% (399)	6% (8)	44% (407)
Sexual health	17% (129)	29% (42)	19% (171)
Social services	13% (98)	33% (47)	16% (145)
Clinical management	6% (46)	6% (9)	6% (55)
Mental health	4% (*n* = 29)	8% (11)	4% (40)
Substance use treatment	3% (27)	7% (10)	4% (37)
Referral management	3% (26)	1% (2)	3% (28)
Employment	2% (12)	8% (12)	3% (24)
Linkage to HIV care	<1% (3)	1% (2)	<1% (5)
Legal/judicial	<1% (3)	0% (0)	<1% (3)
Total	772	143	915

**Table 3:** Summary of Client Perception Coordination Quality and Healthcare Climate Questionnaire by Visit.

	Client Perception Coordination Quality (CPCC) Mean (SD)			Healthcare Climate Questionnaire (HCCQ) Mean (SD)		
	PrEP Users	PrEP Non-Users	*p*-Value ^1^	PrEP Users	PrEP Non-Users	*p*-Value ^1^
Enrollment	-	-		-	-	
Week 4	-	-		6.24 (1.08)	6.09 (1.18)	0.628
Week 8	-	-		6.38 (0.86)	6.15 (1.13)	0.577
Week 13	4.28 (0.69)	4.17 (0.77)	0.171	6.25 (0.95)	6.00 (1.32)	0.616
Week 26	4.33 (0.69)	4.12 (0.74)	0.102	6.19 (1.10)	5.64 (1.52)	0.067
Week 39	4.30 (0.74)	4.25 (0.66)	0.465	6.14 (1.27)	6.04 (1.20)	0.311
Week 52	4.32 (0.76)	4.19 (0.68)	0.060	6.10 (1.30)	5.91 (1.54)	0.648

^1^ Wilcoxon two-sample ranksome test.

**Table 4:** PrEP Continuation.

	OR (95 CI)	*p* Value
Week 4 Healthcare Climate Questionnaire (HCCQ)	1.08 (0.82, 1.45)	0.567
Week 8 Healthcare Climate Questionnaire (HCCQ)	1.48 (1.04, 2.11)	0.031
Week 13 Client Perception Coordination Quality (CPCQ)	0.84 (0.49, 1.44)	0.521

Covariate: site.

## Data Availability

Data may be requested by submitting a Data Request Application Form and a Data Use Agreement to the HPTN Leadership and Operations Center (LOC) at: HPTN@fhi360.org.

## Supplementary Materials

The following supporting information can be downloaded at: https://www.mdpi.com/article/10.3390/ijerph19084489/s1, Full authorship list of the HPTN 073 Study Team.
