# Supplementary material for: Optimizing PrEP Continuance: A Secondary Analysis Examining Perceived Autonomy Support and Care Coordination Quality among Black MSM in HPTN 073
Source: Int J Environ Res Public Health. 2022 Apr 8;19(8):4489. doi: 10.3390/ijerph19084489 (PMC9026517; doi:10.3390/ijerph19084489)
Supplement: Supplementary file 1 [file ijerph-19-04489-s001.zip › ijerph-1599446-supplementary.pdf]

HPTN 073<br>Pre-Exposure Prophylaxis (PrEP) Initiation and Adherence among Black Men who have Sex with Men (BMSM) in Three U.S. Cities<br>PROTOCOL TEAM ROSTER	
**Protocol Chair**<br>Darrell P. Wheeler, PhD, MPH, ASW<br>Dean & Professor<br>Graduate School of Social Work<br>Loyola University Chicago<br>820 N. Michigan Avenue<br>Chicago, IL 60611<br>Phone: (312) 915-7024<br>Email: dwheeler@luc.edu	**Protocol Co-Chair**<br>Sheldon D. Fields PhD, ARNP,<br>FNP-BC, AACRN, DPNAP, FAANP<br>Associate Professor and Assistant Dean of Clinical Affairs and Health Policy College of Nursing and Health Sciences Florida International University<br>11200 SW 8th Street. MMC, AHC3 Room 524<br>Miami, FL 33199<br>Phone: (305) 348-6992<br>Email: sdfields@fiu.edu
David Borasky, MPH, CIP<br>Deputy Director, Office of Human Research Ethics<br>Medical School Bldg 52<br>CB# 7097<br>University of North Carolina at Chapel Hill<br>Chapel Hill, NC 27599-7097<br>Phone: (919) 966-1344<br>Email: dborasky@email.unc.edu	Ying Qing Chen, PhD<br>Full Member & Professor<br>SCHARP/VIDD<br>Fred Hutchinson Cancer Research Center<br>1100 Fairview Ave N, M2-C200<br>Seattle, WA 98109<br>Phone: (206) 667-7051<br>Email: yqchen@fhcrc.org
William Clarke, PhD<br>Johns Hopkins School of Medicine<br>Department of Pathology<br>401 North Broadway, Weinberg 2327<br>Baltimore, MD 21231-2410 USA<br>Phone: (410) 502-7692<br>Email: wclarke@jhmi.edu	Vanessa Elharrar, MD, MPH<br>Medical Officer<br>Prevention Science<br>Program/DAIDS/NIAID/NIH<br>6700-B Rockledge Drive, 5th Fl. Rm. 5249<br>Bethesda, MD 20892<br>Phone: (301) 827-0845<br>Email:elharrarva@niaid.nih.gov
Lynda Emel, PhD<br>Research Program Manager<br>SCHARP/FHCRC<br>1100 Fairview Avenue North<br>E3-129<br>PO Box 19024<br>Seattle, WA 09109 U.S.A<br>Phone: (206) 667-5803<br>Email: lemel@scharp.org	Rebecca Guzmán, CCRP<br>Senior Clinical Research Associate<br>Gilead Sciences, Inc.<br>333 Lakeside Dr., Bldg. 300<br>Foster City, CA 94404<br>Phone: (650) 524-3953<br>Email: rebecca.guzman@gilead.com

Craig Hendrix, MD<br>Johns Hopkins University<br>600 North Wolfe Street<br>Harvey 502<br>Baltimore, MD 21287, U.S.A<br>Phone: (410) 955-9707<br>Email: cwhendrix@jhmi.edu	Lisa Hightow-Weidman, MD, MPH<br>Associate Professor<br>University of North Carolina at Chapel Hill Campus<br>Box 7030<br>Chapel Hill, NC 27599<br>Phone: (919) 843-0033<br>Email: lisa_hightow@med.unc.edu
Stephaun E. Wallace, MS<br>Project Manager<br>Legacy Project Office of HIV/AIDS Network Coordination (HANC)<br>Fred Hutchinson Cancer Research Center<br>1100 Fairview Ave. N, E2-112<br>Seattle WA 98109-1024<br>Voice: 206-667-3108<br>Fax: 206-667-7711<br>Email: sewallac@fhcrc.org	Beryl A. Koblin, PhD<br>Head, Laboratory of Infectious Disease Prevention<br>Member, Lindsley F. Kimball Research Institute<br>New York Blood Center<br>310 E.67th Street<br>New York, NY 10065<br>Phone: (212) 570-3105<br>Email: bkoblin@nybloodcenter.org
Shauna Wolf<br>HPTN LC QA/QC Coordinator<br>Johns Hopkins University School of Medicine<br>600 North Wolfe Street<br>Pathology 304<br>Baltimore, MD 21287, USA<br>Phone: (410) 502-6549<br>Email: swolf14@jhmi.edu	Jonathan Paul Lucas, MPH FHI360<br>Community Program Manager 2224<br>East NC Hwy 54 Durham, North<br>Carolina 27713<br>Phone: (919) 544-7040 x11458<br>Email: jlucas@fhi360.org
Manya Magnus, PhD, MPH<br>Associate Professor<br>Department of Epidemiology and Biostatistics<br>School of Public Health and Health Services<br>George Washington University<br>2100-W Pennsylvania Avenue, NW, 8th floor<br>Washington, DC 20037<br>Phone: (202) 994 3024<br>Email: manyadm@gwu.edu	Cheryl J. Marcus, BA, BSN<br>Clinical Research Director<br>UNC AIDS Clinical Trials Unit<br>130 Mason Farm Road<br>2105 Bioinformatics Building<br>Chapel Hill, NC 27514<br>Phone: (919) 843-8761<br>Email: cjm@med.unc.edu
Kenneth Mayer, MD<br>Infectious Disease Attending and Director of HIV Prevention Research<br>Beth Israel Deaconess Medical Center<br>Visiting Professor of Medicine<br>Harvard Medical School<br>Medical Research Director<br>The Fenway Institute<br>Fenway Health<br>Boston, Mass 02215<br>Phone: (401) 793-4711<br>Email: Khmayer@gmail.com	Anthony Mwatha, MS<br>Biostatistician<br>SCHARP-FHCRC<br>1100 Fairview Ave. North, M2-C200<br>PO Box 19204<br>Seattle WA 98109, USA<br>Phone: (206) 67-7123<br>Email: mwatha@scharp.org

LaRon E. Nelson, PhD, RN, NP<br>Assistant Dean for Global & Community Affairs<br>Assistant Professor of Nursing and Pharmacy<br>University of South Florida, College of Nursing<br>12901 Bruce B. Downs Blvd., MDC 22<br>Tampa, Florida 33612<br>Phone: (813) 974-3351<br>Email: lnelson7@health.usf.edu	Alena V. Pechonkina, MD<br>Clinical Program Manager<br>Gilead Sciences, Inc.<br>Phone: (650) 372-4462<br>Email: alena.pechonkina@gilead.com
Estelle M. Piwowar-Manning, M.T.<br>Johns Hopkins University Hospital<br>Department of Pathology<br>Pathology Room 313<br>600 North Wolfe Street<br>Baltimore MD 21287<br>Phone: (410) 614-6736<br>Email: epiwowa@jhmi.edu	Keith Rawlings, MD<br>Director, Medical Affairs<br>Gilead Sciences, Inc.<br>333 Lakeside Drive<br>Foster City, CA 94404<br>Phone: (650) 372-4463<br>Email: Keith.Rawlings@gilead.com
Rotrease Regan, PhD, MPH, RN<br>Project Director<br>Center for Behavioral and Addiction<br>Medicine Department of Family Medicine<br>David Geffen School of Medicine at UCLA<br>UCLA Vine Street Clinic<br>910 Vine Street<br>Los Angeles, CA 90038<br>Phone: (323) 461-3106<br>Email: rregan@mednet.ucla.edu	James F. Rooney, M.D.<br>Vice President Medical Affairs<br>Gilead Sciences<br>333 Lakeside Drive<br>Foster City, California 94404<br>Phone: (650) 522-5708<br>Email: jim.rooney@gilead.com
Scott Mitchell Rose, BS<br>FHI360<br>Sr. Clinical Research Manager<br>2224 East NC Hwy 54<br>Durham, NC 27713<br>Phone: (919) 405-1447<br>Email: srose@fhi360.org	Katherine Shin, PharmD<br>Pharmaceutical Affairs Branch<br>DAIDS,NIAID.NIH<br>6700-B Rockledge Drive Room 4227<br>Bethesda, MD 20892-7626<br>Phone: (301) 594-1517<br>Email: kashin@niaid.nih.gov

Steve Shoptaw, PhD<br>Professor & Vice Chair Academic Affairs,<br>Dept. of Family Medicine at UCLA<br>10880 Wilshire Blvd, Suite 1800<br>Los Angeles, CA 90095-7087<br>Phone: (310) 794-6206<br>Email: sshoptaw@mednet.ucla.edu	Gregory D. Victorianne, BA<br>Research / Administrative Coordinator<br>Center for Behavioral & Addiction Medicine<br>Department of Family Medicine at UCLA<br>Administrative Office<br>10880 Wilshire Blvd., Suite 1800 Box 957087<br>Los Angeles, CA 90095-7087<br>Phone: (310) 794 0619 ext. 240<br>Email: GVictorianne@mednet.ucla.edu
Steven Wakefield, BS<br>S. Wakefield, Director External Relations<br>HIV Vaccine Trials Network<br>Fred Hutchinson Cancer Research Center<br>1100 Fairview Avenue North, LE-500<br>Seattle, WA 98109-1024<br>Phone: (206) 667-6705<br>Email: wakefield@hvtn.org	Phaedrea Watkins, MBA, CCRP<br>FHI360<br>Clinical Research Manager<br>2224 East NC Hwy 54<br>Durham, NC 27713<br>Phone: (919) 544-7040 x11596<br>Email: pwatkins@fhi360.org
Christopher Chauncey Watson, BS<br>Clinical Research Site Coordinator<br>George Washington Univ. CRS<br>2100-W Pennsylvania Ave, NW<br>Suite 800<br>Washington, DC 20037<br>Phone: (202) 994-3340<br>Email: cclwat@gwu.edu	John K. Williams, M.D.<br>Assistant Professor<br>UCLA, Department of Psychiatry & Biobehavioral Sciences<br>760 Westwood Plaza, 38-260<br>Los Angeles, CA 90024-1759<br>Phone: (310) 825-8810<br>Email: Keoniwmd@aol.com
Leo Wilton, PhD<br>Associate Professor<br>Binghamton University<br>College of Community and Public Affairs (CCPA)<br>Department of Human Development<br>P.O. Box 6000<br>Binghamton, New York 13902<br>Phone: (607) 777-9215<br>Email: lwilton@binghamton.edu	
